# Notes and News

**Published:** 1894-02-24

**Authors:** 


### Notes and News.

Collected and Communicated.

The verdict of medical authorities is looked for with great interest in respect to the definite contagion of phthisis. America has shown an apparent desire to be foremost in establishing preventive measures, yet the Philadelphia Board of Health have refused to declare consumption a contagious disease.

A woman has been very near election as an assistant medical officer to the Workhouse Infirmary, Lambeth. It is significant of the changing opinion of the times, that the lady lost the appointment by but one vote, and that a casting vote by the chairman. Her qualifications must have been admirable indeed, since she lost so narrowly a post which included attendance on both sexes, a proceeding which the most liberal-minded still mostly refuse to deem desirable.

The proposal to establish a hospital for infectious diseases at Chingford is meeting with the usual opposition it is the common fate of all such schemes to encounter. The Walthamstow Local Board have chosen a site bordering one of the roads leading to Epping Forest, which is constantly traversed by naturalists, trippers, and others, and the one objection urged is that these would encounter the patients being conveyed to the hospital, and might thus be the means of introducing fevers from the country into the metropolis when in search of health and pleasure.

Very many members of the medical profession and many old patients of the late Sir Andrew Clark will be sorry to hear that his manservant, Valentine, was attacked with severe bronchitis after the funeral, which caused him much suffering, and has terminated in his death. Valentine was an excellent man in every way, and quite devoted to his master's interests. He leaves a widow, who is a professed cook, and one little girl, whom the mother is anxious to keep with her. Mrs. Valentine desires to obtain (1) a position as cook-housekeeper in a gentleman's or nobleman's family, where she could have her little girl, or (2) the care of chambers or offices. We should be glad to hear from any one who may be able to help Mrs. Valentine to secure the position she seeks.

The Harness action against *Truth* is over, for Mr. Harness has withdrawn. Reasonable people will be able to judge for themselves on the evidence of the case. The foolish cannot be saved from themselves.

The Croonian Lecture of the Royal Society for 1894 will be delivered by S. Ramon Y. Cajal, Professor of Histology and Pathological Anatomy in the University of Madrid. The subject will be the "Minute Scructure of the Nervous System," and the date March 1st.

Facts elicited at the sitting of the Opium Commission at Bombay, on the 16th inst., will not help the anti-opium cause. One petition against opium contained seventy-three signatures of persons purporting to be opium smokers. Investigation of fifty-four cases showed that only three of these were genuine.

The Manchester Royal Infirmary and Owens College have cemented their relationship. At the annual meeting of the infirmary, beds were granted to the professors of systematic medicine and surgery, in order that the college authorities, when electing to vacant chairs, should not be limited in their choice to members of the medical staff of the infirmary.

The new medical school at Cardiff was opened on the 14th inst., Lord Windsor presided. Sir Richard Quain delivered the inaugural address, which consisted of a survey of medical education in general, and of its rise, progress, and present condition at Cardiff in particular. The medical school is in connection with the University College of South Wales and Monmouthshire. Eighteen thousand pounds is wanted to found the school on proper lines, and towards this £600 has already been subscribed.

There was a last-century air about the discussion raised at the annual meeting of the Bedford General Infirmary, on the method of electing the house surgeon. It will amaze many readers to hear that for ninety years and more house surgeons at Bedford have been elected by the whole body of governors. We should have imagined that the expense of such a Parliamentary poll in miniature would have caused the abolition of so obsolete a system long ago. Far from this being the case, however, Mr. Sharpin, who is apparently a crusted Tory, contended that abolition would in this case mean financial disaster. He contended that many governors subscribed £2 2s. to get a vote, where they would only give one for charity's sake. He further contended if the weekly committee at Bedford were charged with the duties of electing the officers of the infirmary they would become virtually the nominees of the medical officers. Such arguments prove the desirability of abolishing the old bad system at Bedford in favour of election by the Committee. We counsel the Governors to immediately pass new regulations empowering the weekly Board to elect all officers, and so to bring themselves into line with the best administered hospitals throughout the country. The old bad system of election must have tended to exclude candidates of independent spirit and ability, who would naturally decline to expend large sums of money and time upon a canvass for a post which ought to be given as a matter of course to the best qualified candidate apart from every other consideration whatever.

